# Case Report: Novel *FOXC1* variant c.311T>G (p.Ile104Ser) in a Chinese family with Axenfeld-Rieger syndrome

**DOI:** 10.3389/fmed.2026.1868263

**Published:** 2026-06-08

**Authors:** Bin Lin, Li Li, Dong-kan Li

**Affiliations:** 1 Xiamen Eye Center and Eye Institute of Xiamen University, School of Medicine, Xiamen, China; 2 Xiamen Clinical Research Center for Eye Diseases, Xiamen, Fujian, China; 3 Xiamen Key Laboratory of Ophthalmology, Xiamen, Fujian, China; 4 Fujian Key Laboratory of Corneal & Ocular Surface Diseases, Xiamen, Fujian, China; 5 Xiamen Key Laboratory of Corneal & Ocular Surface Diseases, Xiamen, Fujian, China; 6 Translational Medicine Institute of Xiamen Eye Center of Xiamen University, Xiamen, Fujian, China

**Keywords:** Axenfeld-Rieger syndrome, *FOXC1*, incomplete penetrance, juvenile-onset open-angle glaucoma, novel variant, whole-exome sequencing

## Abstract

**Background:**

Juvenile-onset open-angle glaucoma (JOAG) is a heterogeneous early-onset glaucoma subtype. Axenfeld-Rieger syndrome (ARS) is an autosomal dominant disorder caused by *FOXC1* variants, which may present with severe early-onset glaucoma and can be clinically mistaken for JOAG. This study aimed to determine the genetic cause and clarify the clinical diagnosis in a Chinese family initially diagnosed with JOAG.

**Methods:**

A 15-year-old male proband and his family members received detailed ophthalmic and systemic evaluations. Whole-exome sequencing was performed in the proband, and candidate variants were verified by Sanger sequencing. Bioinformatic tools were used to evaluate variant pathogenicity.

**Results:**

The proband and his elder sister initially presented with severe early-onset glaucoma and were diagnosed with JOAG at an outside hospital. After systemic evaluation, they were found to have anterior segment dysgenesis and characteristic facial features, including midface hypoplasia, hypertelorism, and saddle nose deformity, supporting a revised diagnosis of ARS. A novel heterozygous missense variant c.311T>G (p.Ile104Ser) in *FOXC1* was identified. This variant was absent from the East Asian population in gnomAD and was predicted to be highly deleterious. It co-segregated with the disease phenotype and showed incomplete penetrance in the unaffected father.

**Conclusion:**

We identified a novel FOXC1 variant responsible for ARS in a Chinese family. ARS-associated glaucoma can closely mimic JOAG, leading to initial misdiagnosis. The observation of incomplete penetrance in an unaffected carrier father underscores the phenotypic complexity of this disorder. Our findings expand the genotypic spectrum of FOXC1-related disorders and highlight the importance of systemic evaluation and genetic testing in patients with early-onset glaucoma.

## 1 Introduction

Juvenile-onset open-angle glaucoma (JOAG) is a subtype of primary open-angle glaucoma (POAG) with an onset age before 40 years. The disease can be familial or sporadic, and the proportion of familial and sporadic cases varies across populations (1). Clinically, JOAG is characterized by elevated intraocular pressure (IOP), and many patients require surgical intervention due to poor response to medical therapy. Similar to other forms of glaucoma, the core pathological process of JOAG involves progressive loss of retinal ganglion cells, and elevated IOP is recognized as a major but not exclusive causative factor. The underlying pathophysiological mechanisms of glaucoma are diverse and remain incompletely understood in most cases (2, 3).

Regarding genetics, variants in multiple genes contribute to the development and progression of glaucoma. For instance, *MYOC* (myocilin) mutations represent the most common genetic cause of JOAG. Variants in the *FOXC1* gene (forkhead box C1) also frequently induce glaucoma in adolescents, and these two etiologies are easily confused clinically, although their underlying pathogenic mechanisms may differ. To date, pathogenic variants in *FOXC1* have been widely documented in the Human Gene Mutation Database (HGMD), including nonsense, frameshift, splicing, and missense variants. However, the genotype–phenotype correlation is complex, and an identical variant can lead to highly variable clinical manifestations across families or even within the same family, posing challenges for genetic counseling and clinical management.

The *FOXC1* gene encodes a pivotal transcription factor that regulates the development of the anterior segment of the eye, heart, and craniofacial region. Heterozygous pathogenic variants in *FOXC1*cause autosomal dominant Axenfeld-Rieger syndrome (ARS) type 3 (4, 5). The clinical spectrum of *FOXC1*-related disorders is broad, ranging from classic ARS (with systemic features such as iris hypoplasia, corneal dystrophy, and dental anomalies) to non-syndromic congenital or early-onset open-angle glaucoma (6, 7). Although *FOXC1* plays a critical role in ocular development and anterior chamber angle formation, its mutations are more commonly associated with ARS, and patients with ARS frequently develop secondary juvenile-onset glaucoma.

Herein, we report a Chinese glaucoma family in which the proband and his elder sister presented with severe glaucoma. At the same time, their father, who carried the same variant, exhibited a normal clinical phenotype. Using whole-exome sequencing, we identified a novel missense variant c.311T>G (p.Ile104Ser) in the *FOXC1* gene in this family, and analyzed and discussed its clinical significance.

## 2 Case report

### 2.1 Clinical information

The patient, a 15-year-old male, was referred to our hospital with a 2-year history of right eye distension and significant visual loss. He denied obvious redness, pain, photophobia, or epiphora. He had been diagnosed with juvenile-onset open-angle glaucoma (JOAG) in the right eye at another hospital but showed poor response to topical hypotensive medications; he was therefore referred to our clinic for further etiological evaluation. The patient had a history of trauma in the left eye, which resulted in retinal detachment. No further intervention was performed due to no light perception in the left eye.

Ophthalmic examinations revealed:

*Visual acuity*: light perception in front of the right eye, and no light perception in the left eye.

IOP: 36.6 mmHg in the right eye (after using three anti-glaucoma medications), and 15.3 mmHg in the left eye.

Slit-lamp examination showed mild corneal edema, deep anterior chamber, sparse iris texture, round pupil, and transparent lens in the right eye. In the left eye, there was band keratopathy, shallow anterior chamber, posterior synechiae of the iris, and total cataract, with no view of the fundus. B-scan ultrasonography confirmed old retinal detachment in the left eye.

**Figure 1:**
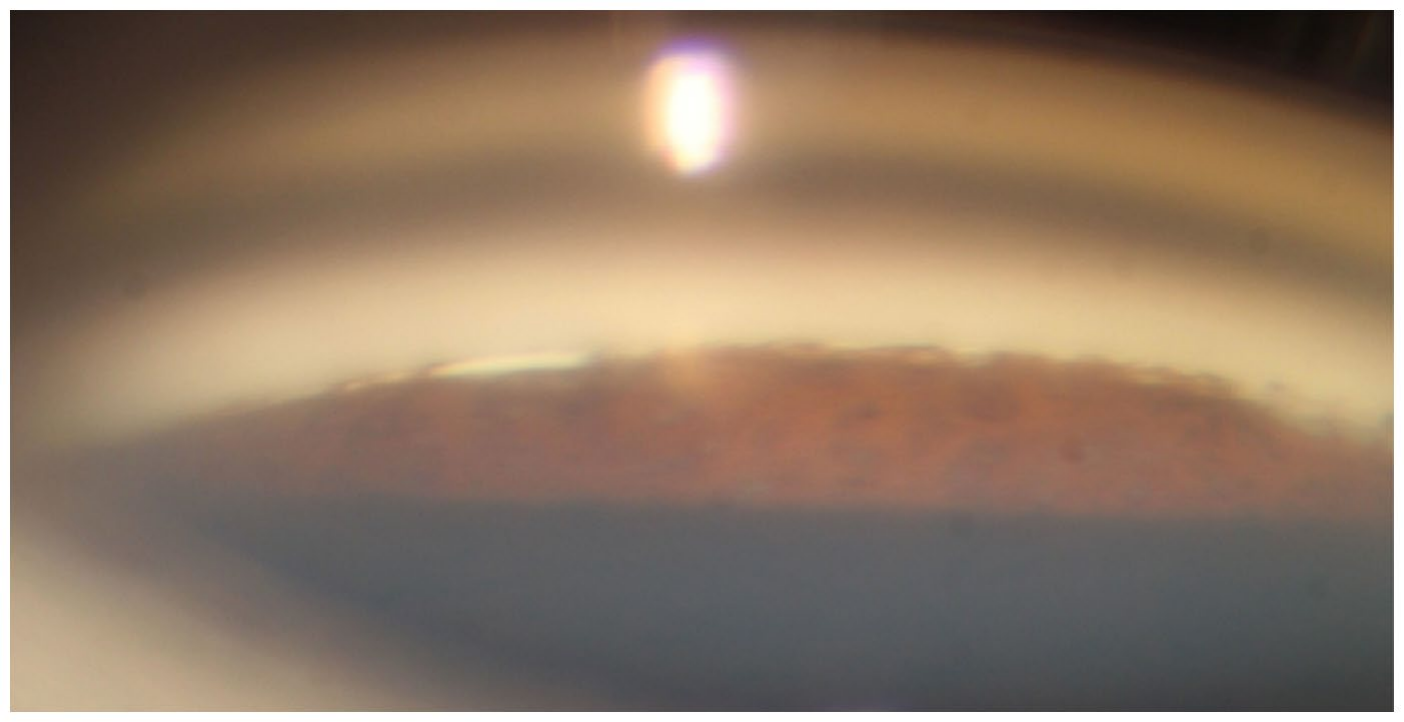
Gonioscopic findings of the patient. The view is hazy due to corneal edema; however, high iris root insertion and increased pigmentation of the trabecular meshwork are still visible.

Gonioscopy revealed dysgenesis of the anterior chamber angle in the right eye. Although visualization was impaired by corneal edema, a wide-angle, high-iris root insertion, and increased pigmentation of the trabecular meshwork could still be observed (Figure 1).

These findings led us to suspect that the patient did not have isolated JOAG.

**Figure 2:**
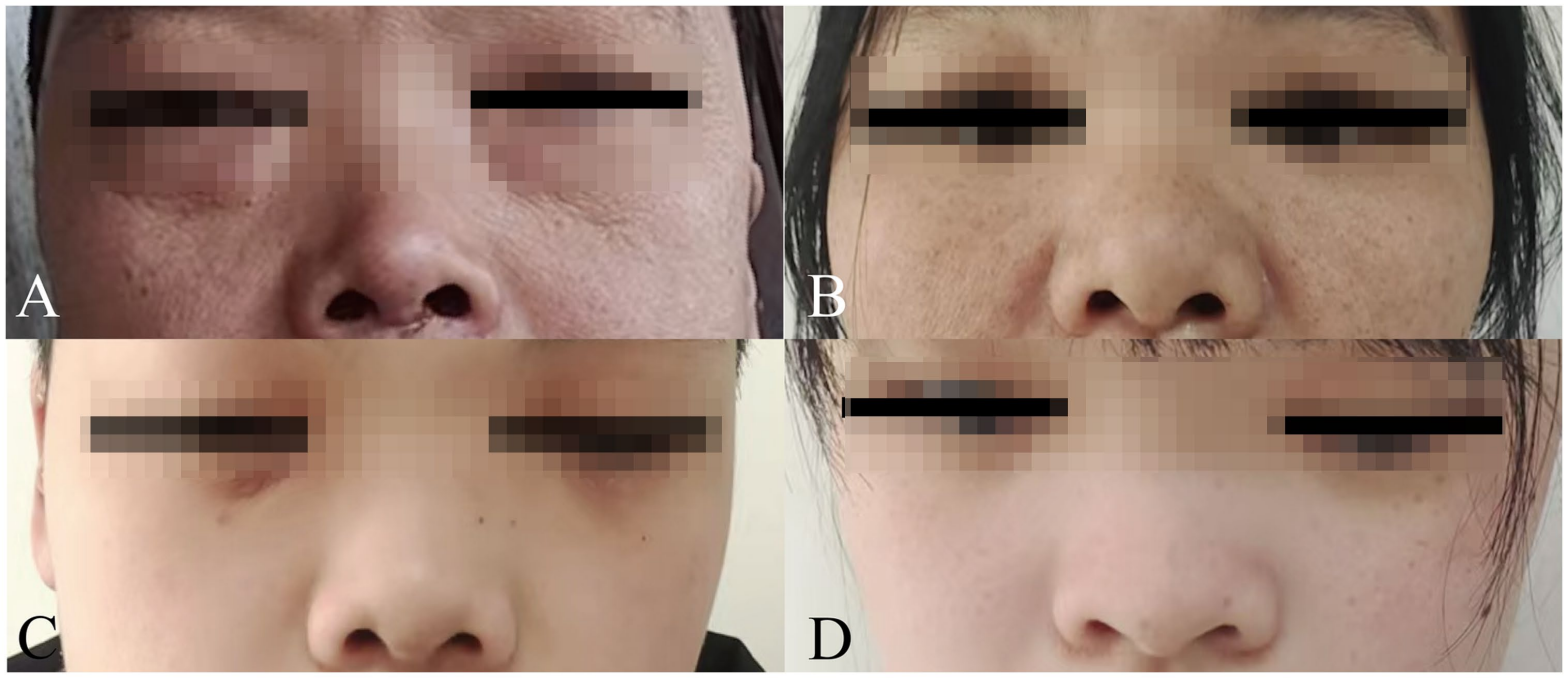
Facial features of the four family members. **(A)** Father; **(B)** mother; **(C)** the proband (son); **(D)** daughter. Characteristic midface hypoplasia, hypertelorism (increased intercanthal distance), and saddle nose deformity can be observed.

During the consultation, we further observed the patient’s systemic and facial characteristics, as well as those of his family members. Systematic examination revealed distinctive midface dysplasia in the family, including midface hypoplasia, hypertelorism (increased intercanthal distance), and saddle nose deformity. The detailed facial features are shown in Figure 2.

Family investigation showed that the proband’s biological elder sister was also diagnosed with bilateral glaucoma. Her visual acuity was light perception in both eyes, and the intraocular pressures were 25 mmHg and 28 mmHg in the right and left eyes, respectively. She denied any other systemic abnormalities. And she was prescribed a fixed combination of brinzolamide and brimonidine eye drops (twice daily) to lower her intraocular pressure. Due to personal and financial considerations, she has not undergone further surgical intervention or long-term follow-up at our institution to date. The proband’s parents denied a history of glaucoma or visual impairment. Detailed ophthalmic examinations revealed no obvious abnormalities in intraocular pressure, visual acuity, anterior segment, or fundus in either parent, and no typical dysgenesis of the anterior chamber angle was observed. No other family members were reported to have similar ocular diseases.

Finally, based on ocular manifestations, facial dysmorphic features, and a familial pattern, we strongly suspected a clinical diagnosis of Axenfeld-Rieger syndrome (ARS). We planned to perform targeted genetic testing for this family.

### 2.2 Genetic testing and results

After obtaining written informed consent from all participants (the proband, his parents, and his elder sister), peripheral blood samples were collected for genetic analysis. Whole-exome sequencing was performed on the proband, and candidate variants were verified in the family using Sanger sequencing. Of note, due to privacy concerns, the parents declined genetic testing for other extended family members beyond the nuclear family; thus, genotyping data from additional relatives were not available.

**Figure 3:**
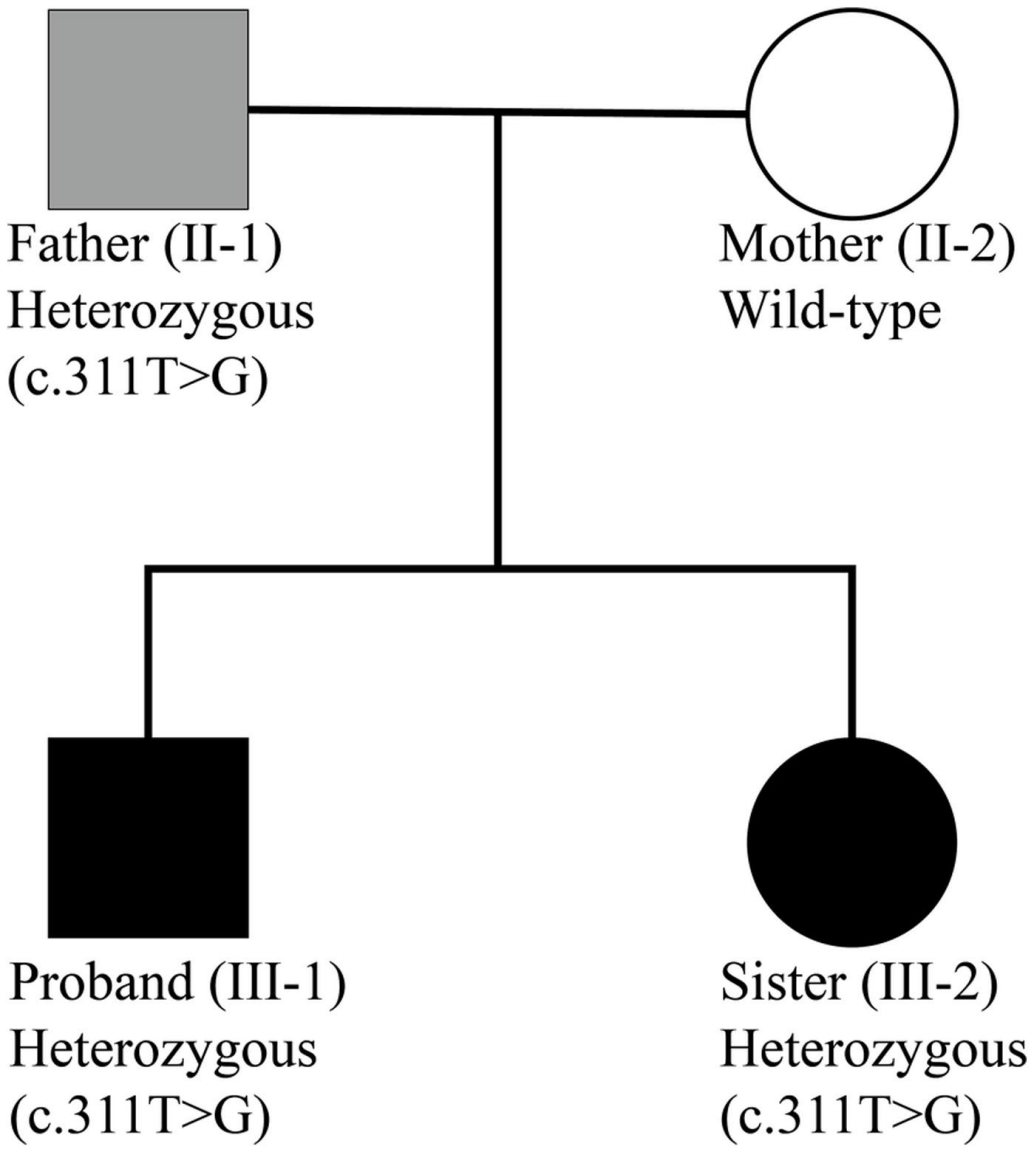
Pedigree of the affected family. The father, the proband (son), and the daughter all carry the same genetic variant. The father is shown in gray (asymptomatic carrier with normal phenotype), while the son and daughter are shown in black (clinically affected with ocular and facial manifestations).

Sequencing results revealed that the proband carried a heterozygous missense variant in exon 3 of the *FOXC1* gene (NM_001453.3): c.311T>G, which results in the substitution of isoleucine by serine at codon 104 (p.Ile104Ser). The same variant was identified in his elder sister and father (Figure 3). This variant was absent from the East Asian population in gnomAD, indicating it is extremely rare. According to the guidelines of the American College of Medical Genetics and Genomics (ACMG) (8, 9), the variant was initially classified as a variant of uncertain significance (VUS).

Multiple bioinformatic tools predicted that this variant is deleterious to gene or protein function, with a REVEL score of 0.975. Furthermore, using AlphaMissense (DeepMind), a state-of-the-art method published in Nature in 2026 (9), this variant was classified as Pathogenic Strong with a perfect score of 1.0. According to the original publication, AlphaMissense thresholds are defined as follows: < 0.340 for Likely benign and > 0.564 for Likely pathogenic, with scores in between indicating ambiguous significance. Thus, this variant achieved the maximum deleterious score.

**Figure 4:**
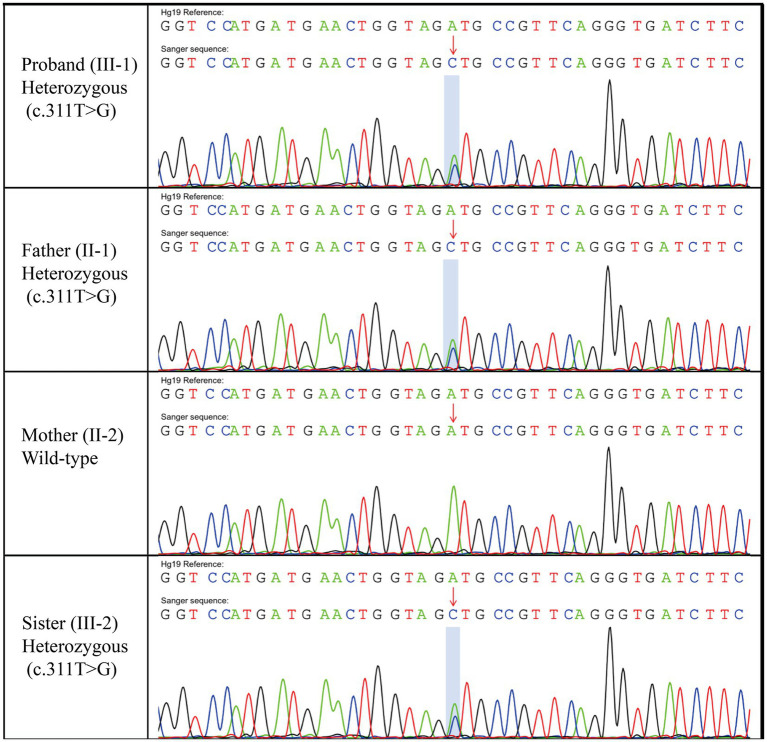
Pedigree Sanger sequencing chromatograms. The same variant was detected in the proband, his affected elder sister (III-2), and his phenotypically normal father (II-1). The phenotypically normal mother (II-2) showed a wild-type sequence at this locus.

Pedigree validation by Sanger sequencing (Figure 4) showed that the variant was heterozygous in the affected elder sister (III-2) and the phenotypically normal father (II-1). In contrast, the phenotypically normal mother (II-2) did not carry it. The variant’s transmission pattern in this family was consistent with autosomal dominant inheritance.

To further exclude other potential pathogenic causes, we analyzed the *PITX2* variant (c.197A>G) that was also detected in the proband. Although *PITX2* is a known cause of ARS (10) and its variants can exhibit incomplete penetrance (11, 12), segregation analysis revealed that the *PITX2* c.197A>G variant was absent in the affected elder sister. According to the ACMG/AMP guidelines (13), the lack of segregation of a variant with the disease phenotype in an affected family member provides strong evidence against its pathogenicity (criterion BS4). Therefore, this *PITX2* variant is considered unlikely to be the primary disease-causing variant in this family and was classified as an incidental or secondary finding.

## 3 Discussion

In this report, we identified a novel heterozygous missense variant c.311T>G (p.Ile104Ser) in the FOXC1 gene in a Chinese family with autosomal dominant glaucoma. The variant is absent from the East Asian subpopulation in gnomAD, indicating it is extremely rare in the general population. Furthermore, to the best of our knowledge, this specific variant has not been previously reported in the literature in any ethnic group. The combination of its population rarity, absence in published reports, strong deleterious predictions by multiple bioinformatic tools, and high consistency with the clinical phenotype of ARS spectrum disorders strongly supports its potential pathogenicity.

Our case provides critical clinical and genetic evidence for reclassifying this VUS. The variant demonstrated clear familial segregation with the ARS phenotype: it was present in the two affected siblings (proband and his elder sister) and absent in the unaffected, non-carrier mother, consistent with autosomal dominant inheritance. Notably, the variant was also identified in their clinically unaffected father, demonstrating incomplete penetrance—a feature of *FOXC1*-related disorders (14–16). Furthermore, the affected siblings exhibited a classic *FOXC1*-related phenotypic spectrum, including glaucoma, anterior chamber angle dysgenesis, and characteristic systemic features (e.g., saddle nose, hypertelorism). The congruence of this specific phenotype with the variant’s location and its *in silico* predicted deleterious effect strengthens the pathogenicity argument.

Another important finding in this family is the illustration of variable expressivity. The significant differences in disease severity and associated ocular complications between the proband and his elder sister, despite carrying the identical *FOXC1*variant, highlight the substantial phenotypic variability that can exist even within a single family. This variability, coupled with the observed incomplete penetrance in the father, underscores the complex clinical heterogeneity of *FOXC1*-related disorders. The mechanisms underlying this heterogeneity are likely multifactorial, potentially involving modifier genes, epigenetic regulation, or stochastic developmental events. These findings enrich our understanding of the clinical heterogeneity of *FOXC1*-related disorders. Clinically, this reinforces the need for comprehensive and lifelong ophthalmic surveillance in all carriers of a *FOXC1* variant, regardless of their current symptomatic status or the severity of disease in their relatives.

Clinically, this case highlights the value of genetic testing in the diagnosis and management of congenital glaucoma. Even if parents have normal clinical phenotypes, the risk of autosomal dominant ocular disease in offspring cannot be excluded. Therefore, once a *FOXC1* variant is identified in a proband, targeted genetic testing and systematic ophthalmic screening should be performed for all first-degree relatives, which are crucial for early detection and intervention in asymptomatic carriers. Meanwhile, it provides clear genetic counseling and reproductive guidance for family members.

In summary, although the *FOXC1* c.311T>G variant is novel in public databases, we conclude that it is highly likely pathogenic based on its co-segregation in the family, strong deleterious bioinformatic predictions, and precise matching with the ARS phenotype. This case provides important clinical evidence for the pathogenicity of this VUS and underscores the need for long-term follow-up and functional studies for such variants.

This study has several limitations. First, genotyping data from a larger extended family were not available. Second, no functional assays (such as reporter gene assays or protein subcellular localization analysis) were performed to directly confirm the impairment of *FOXC1* protein function by this variant. Finally, the precise molecular mechanism underlying the father’s asymptomatic status (e.g., protective modifier factors) remains unclear and warrants further investigation.

## 4 Conclusion

We report a Chinese glaucoma family harboring a novel missense variant c.311T>G (p.Ile104Ser) in *FOXC1*. This variant co-segregates with the disease phenotype in the family and is predicted to be deleterious by bioinformatic analysis. The observations of incomplete penetrance (asymptomatic carrier father) and variable expressivity (phenotypic differences between the siblings) in this family expand our understanding of the phenotypic diversity of *FOXC1*-related disorders. Our findings support the classification of *FOXC1* c.311T>G as a likely pathogenic variant and underline the critical importance of genetic testing and systematic familial screening in similar clinical settings.

## Data Availability

The datasets presented in this study can be found in online repositories. The names of the repository/repositories and accession number(s) can be found in the article/supplementary material.
